# Investigation of endocrine and cerebral response and nutrition and physical performance parameters according to bigorexia nervosa levels: a cross-sectional study in sports sciences faculty students

**DOI:** 10.1038/s41598-025-27706-2

**Published:** 2025-11-24

**Authors:** Ali Ozan Erkılıç, Bülent Bayraktar, Tuğçe Orkun Erkiliç, Mutlu Türkmen, Murat Kul, Mehmet Yönal

**Affiliations:** 1 Faculty of Sports Sciences, Department of Recreation, Bayburt University, 69000 Bayburt, Turkey; 2 Faculty of Health Sciences, Department of Physiotherapy and Rehabilitation, Bayburt University, Bayburt, Turkey; 3 Department of Nutrition and Dietetics, Faculty of Health Sciences, Bayburt University, 69000 Bayburt, Turkey; 4 Faculty of Sports Sciences, Department of Coach Education, Bayburt University, 69000 Bayburt, Turkey; 5 Gazi University Rectorate, Ankara, 06000 Turkey

**Keywords:** Bigorexia nervosa, 6-Minute walk test, Metabolic equivalent, Body mass index, Asprosin, Brain derived neurotrophic factor, GLP-1, Hormone, Diseases, Endocrinology, Health care, Medical research, Physiology, Risk factors

## Abstract

The aim of this study is to investigate the effects of Bigorexia nervosa (BGN) levels on endocrine (Asprosin, GLP-1) and cerebral (BDNF) responses and nutritional and physical (6MWT, Six-Minute Walk Test)), speed, metabolic equivalent unit (MET)) performance parameters in students of the faculty of sports sciences. This study investigated muscle dysmorphic disorder (MDD), physical activity levels, and specific hormonal markers in 120 university students (63 females, 57 males) aged 18–25 years, all studying sport sciences and reporting no existing health problems. Participants completed a demographic questionnaire, food frequency questionnaire and the Muscle Dysmorphic Disorder (Bigorexia) Inventory through face-to-face interviews. Physical activity was assessed using the 6-Minute Walk Test (6MWT) to measure walking distance, and Metabolic Equivalent (MET) was calculated based on the American College of Sports Medicine (ACSM) formula. Body Mass Index (BMI) was determined from anthropometric measurements of height and body weight. Saliva samples were collected to analyze Asprosin, BDNF, and GLP-1 hormone levels using the ELISA technique.Data were analyzed using number, percentage, mean, chi-square, t-test, Pearson correlation, and ANOVA tests. In all analyses, significance was accepted as *p* < 0.05. According to the demographic findings of the study, the majority of participants (64.2%) were third-year students, and 52.5% were female. The mean age of the participants was 22.01 years, the mean height was 168.59 cm, and the mean weight was 62.61 kg. 73.3% of the individuals were classified as normal weight, 12.5% as pre-obese, 11.7% as underweight, and 2.5% as obese. Men had significantly higher bigorexia and its subscale scores than women (*p* < 0.05), and bigorexia was positively correlated with height and negatively correlated with BMI (*p* < 0.01). Mean salivary BDNF levels were higher in participants consuming 1–2 meals per day (*p* = 0.035), and mean salivary asprosin levels were significantly higher in the obese group compared to the healthy group (*p* = 0.008). Additionally, a strong positive correlation was found between asprosin and GLP1 levels (*r* = 0.585; *p* < 0.01). Bigorexia status was significantly associated with meat, meat products, and fat consumption (*p* < 0.05), while no association was found with physical performance parameters such as 6MWT, speed, and MET (*p* > 0.05). The average MET value was found to be 4.53, indicating that participants generally engaged in moderate-intensity physical activity. In conclusion, examining endocrine (asprosin, GLP-1) and cerebral (BDNF) hormone responses, as well as nutritional and physical performance parameters, according to BGN levels reveals the impact of BGN on physiological and nutritional behaviors. Given the impact of BGN trends on students’ health, examining hormone profiles and their relationships with physical performance and nutrition is believed to significantly contribute to understanding the health problems associated with BGN and developing appropriate interventions.

## Background

 Bigorexia nervosa (BGN) is a body dysmorphic disorder characterized by an obsession with increasing muscle mass and decreasing fat^1,2^.While its prevalence varies widely worldwide, ranging from 1% to 54%, rates increase significantly in certain groups; for example, it is estimated to be approximately 10% in bodybuilders and gym members, 12.7% in soldiers^3,4^ and is less common in women at 4.2%^4^. Rates of bigorexia among university students have been found to range from 1.3% to 6.6%^5,6^ and these rates are particularly high in individuals using anabolic steroids^7^.According to the American Psychiatric Association’s DSM-5, bigorexia is a subtype of body dysmorphic disorder, falling under the supercategory of Obsessive-Compulsive and Related Disorders^8^.Due to this preoccupation with muscles, individuals with bigorexia may develop unhealthy eating habits, such as excessive protein consumption, fat and carbohydrate restriction, strict diets, and dependence on nutritional supplements^1,9^.

Appetite control is governed by the hypothalamus in the brain. The lateral hypothalamic area (LHA) within the hypothalamus functions as the hunger center, while the ventromedial hypothalamus (VHM) functions as the satiety center^10^.Signals secreted from the intestine, pancreas, and adipose tissue are transmitted to the hypothalamus. Hormones are important biomolecules that affect all physiological systems, including appetite and emotional state^11^.Adipokines secreted from adipose tissue play a role in many physiological processes such as metabolism, appetite and energy balance^2,12^.As an orexigenic (appetite-increasing) and glucogenic adipokine, asprosin increases glucose synthesis and/or release in the liver and stimulates appetite in the hypothalamus by activating the cAMP signaling pathway through an unknown G protein-coupled receptor^13^.Bigorexia, which involves an obsessive focus on building muscle mass, can be a constant source of stress for individuals, and stress can negatively impact brain chemistry and function, thereby negatively impacting behavior and mood^14^.Brain-derived neurotrophic factor (BDNF), a neurotrophic factor involved in neuronal development, survival, differentiation, and plasticity also regulates neurogenesis in specific brain regions, including the subventricular zone and dentate gyrus^15^ and plays a key role in multiple stress-related mental and psychological disorders^14^.Glucagon-like peptide-1 (GLP-1), an insulinotropic incretin secreted from L cells in the small intestine in response to food intake and causing glucose-dependent insulin secretion^16^ has its primary source in the brain from preproglucagon neurons in the nucleus tractus solitarii and intermediate reticular nucleus in the lower brainstem^17^. In addition to being a potent regulator of food intake, GLP-1 has proliferative and protective effects on islet cells and inhibits gastric emptying. It also has an important physiological role in the brain in response to stress^18,19^.

The 6-Minute Walk Test (6MWT), which reflects activities of daily living, is performed with submaximal effort, and is considered the “Gold Standard” of exercise capacity by measuring functional capacity. It is an easy-to-use and inexpensive physical performance test that examines the responses of many systems such as pulmonary, cardiovascular, circulatory, neuromuscular, and muscle metabolism by assessing the maximum distance covered in 6 min^20,21^. (Boucault et al., 2013; Dalgas et al., 2012). MET (Metabolic Equivalent) is a unit that indicates how much energy an activity expends compared to rest. In psychological disorders, the 6MWT and MET levels are generally used to assess physical activity level and its impact on mental health^22^. To address the gap in the existing literature, our study aimed to investigate the physiological mechanisms underlying BGN. In this context, markers such as asprosin, GLP-1, and BDNF are closely related to metabolism, appetite regulation, anxiety, and neurological responses, which are key components of BGN. Specifically, asprosin (an adipokine that stimulates appetite and promotes gluconeogenesis) and GLP-1 (an incretin that suppresses appetite) were selected because they influence adipokines and incretins that regulate appetite and metabolism through the dietary restrictions, high protein intake, and obsessive changes in body composition characteristic of BGN. Investigating the relationship between these hormones and BGN-related unhealthy eating behaviors and changing BMI is critical for understanding the metabolic implications of BGN. Additionally, the direct relationship of BDNF to neuroplasticity, cognitive function, and stress/anxiety disorders was considered. Because BGN is associated with severe body dysmorphia and anxiety disorders, we aimed to investigate the neurological responses to the disorder by examining the potential effects of obsessive exercise and mental stress on BDNF levels in individuals with BGN.No studies have been found examining the relationship between BGN levels, a common condition among athletes, and endocrine (asprosin, GLP-1) and cerebral (BDNF) levels. In this study, we hypothesize that there will be an association with increased levels of Bigorexia Nervosa (BGN) and increased levels of the endocrine markers Asprosin and GLP-1, decreased levels of cerebral response BDNF, and lower physical performance parameters (6MWT, speed, and MET).

## Methods

### Participants and procedures

**Fig. 1:**
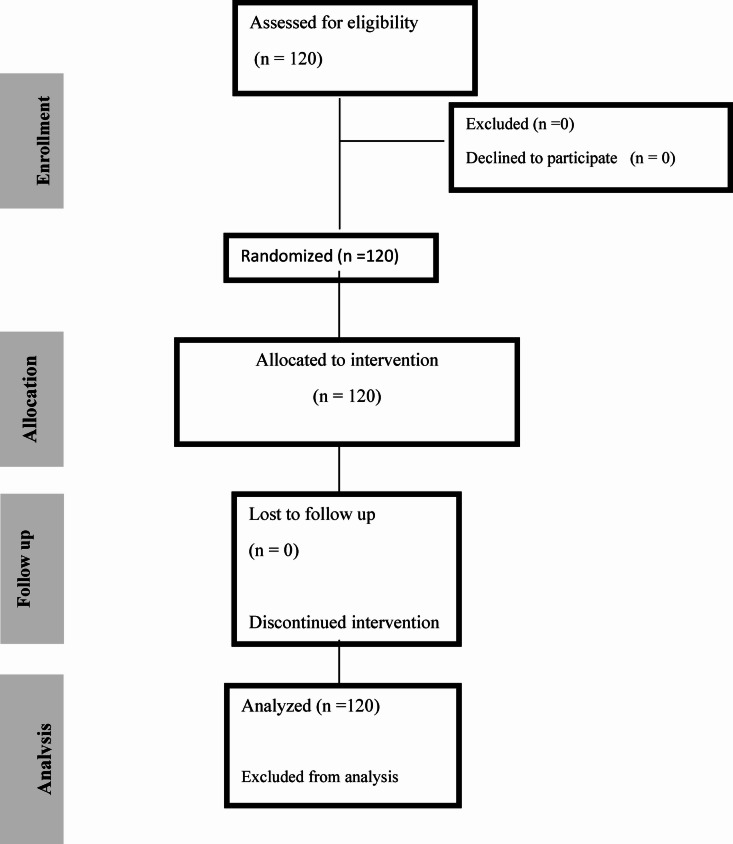
Study cohort flow chart.

The study population consisted of healthy university students between the ages of 18–25 studying in Türkiye. The sample size of the study was calculated using G*Power 3.1.9.7 analysis program; it was determined as 120 with a 95% confidence interval, 5% margin of error, and 80% power. Data were collected using a general information form for university students and the Muscle Dysmorphic Disorder (Bigorexia) Inventory via face-to-face interviews lasting an average of 15 min. In addition, saliva samples were taken from the individuals included in the study to determine asprosin, BDNF, and GLP-1 hormone levels. For this purpose, the study was conducted on a total of 120 university students between the ages of 18–25 studying at Bayburt University Faculty of Sports Sciences. Prior to the study, ethics committee approval (12.12.2024/Decision no: 95) and permission from the Non-Interventional Clinical Research Ethics Committee Commission were obtained. Participants were informed about the study in accordance with the Declaration of Helsinki, and their informed consent was obtained by filling out the Informed Consent Form. Volunteer participants were included in the study (Fig. 1).

### Measures

Data were collected from university students (18–25 years of age) face-to-face using a Personal Information Form and the Muscle Dysmorphic Disorder (Bigorexia) Inventory, which took an average of 15 min. Saliva samples were collected from the athletes. Participating children were instructed not to drink liquids, not to eat, and not to brush their teeth 1 h before saliva collection. They were instructed to avoid tryptophan-rich foods such as red meat, eggs, fish, nuts, seeds, and yogurt, and to avoid brushing their teeth. They were also instructed to keep a detailed food frequency questionnaire before saliva collection. Saliva samples were collected between 8:00 and 9:00 AM using passive saliva collection, in 5 cc aliquots of Salivette tubes (Sarstedt, GERMANY). After being centrifuged at 2000 g for 20 min in a refrigerated centrifuge (NF 1200R, NÜVE, Ankara, TÜRKİYE) in the laboratory, saliva samples were stored at −80 °C until analysis of apelin, asprosin, BDNF and GLP-1 hormone levels.

*Measurement of asprosin*,* BDNF and GLP-1 hormone levels in salivary*: The commercial kits used in measurements are human-specific; Human Asprosin ELISA Kit.” (BT LAB, Cat. No. E4095 Hu, China), Human Brain-derived Neurotrophic Factor ELISA Kit (BT LAB, Cat.No E1302Hu, CHINA), Human Glucagon-like peptide ELISA Kit, Product code: E0022Hu, CHINA) was used. It was studied in accordance with the procedure specified in the manufacturer’s catalog, using an intra-assay coefficient of 8.0% and an inter-assay coefficient of 10.0%. The results were evaluated by reading absorption values at 450nm in accordance with the procedure reported in the kit.

#### Muscle dysmorphic disorder (Bigorexia) inventory

The muscle dysmorphic disorder (bigorexia) inventory was used in the study to measure the level of bigorexia in athletes. The Turkish adaptation of the inventory, developed by Hildebrant et al.^23^, was conducted by Devrim^24^.This scale, which does not include a reverse item, is a 5-point Likert-type scale consisting of 13 items. The scale includes items such as “never (1), sometimes (2), undecided (3), often (4), and always (5). Hildebrant et al.^23^ adopted a cutoff point of 39 for the scale. A score above 39 indicates that individuals have a high level of bigorexia. The Cronbach’s alpha reliability coefficient for the Bigoreksiya scale was determined to be 0.854.

*The 6-minute walk test (6MWT)*: 6MWT was calculated using the Enright-Sherrill Formula based on age, gender, and body mass index^25^. A common formula for calculating the expected 6MWT distance (in meters) for men is: Distance (_m_) = (7.57 × height _cm_) − (5.02 × age _years_) − (1.76 × weight _kg_) − 309.

A common formula for calculating the expected 6MWT distance (in meters) for women is:

Distance (_m_) = (2.11 × height _cm_) − (2.29 × weight _kg_) − (5.78 × age _years_) + 667.

#### MET (Metabolic Equivalent) level

MET is another variable that can be estimated by the 6MWT through a calculation that converts 6-min walking speed into a MET level ([3.5 + (6MWTspeed × 0.1)]/3.5)^26,27^.

### Data analysis

Data obtained through survey forms in the study were processed and analyzed by the researcher using the SPSS 26.0 package program. As a result of the analysis performed for the normality test of the data, the skewness and kurtosis (Skewness and Kurtosis) values for all scales and their sub-dimensions were found to be between − 2 and + 2, and the normality assumption was accepted. For demographic variables such as gender, BMI, and number of meals consumed per day, parametric tests were used for two-group comparisons, and ANOVA tests were used for comparisons of more than two groups. For the two-group variables of gender and number of meals, an independent samples t-test was used, and for the BMI variable with more than two groups, ANOVA analyses were used. Groups with significant differences were determined using Tukey’s HSD, a post-hoc test. Cronbach’s alpha analysis was performed to determine the reliability of the scales and their sub-dimensions. Cronbach’s alpha value is expected to be above 0.70^28^.Pearson correlation analysis was applied to examine the relationships between the scale and its subdimensions used in the study, as well as the BDNF, Asprosin, and GLP1 measurements, and age, height, weight, and BMI. Chi-square analyses were used to examine the relationships between participants’ bigorexia status and their product consumption in the last month. The significance (p) value was set at 0.05 in all analyses. In the applied test results, differences were considered statistically significant when *p* < 0.05, and statistically insignificant when *p* > 0.05.

## Results

According to the demographic findings of the study, 52.5% of the participants were female (*n* = 63) and 47.5% were male. The majority of the participants (64.2%) were third-year students. When the number of daily meals was examined, it was determined that they most frequently consumed 3 meals (46.7%) and 2 meals (40.8%). Based on BMI groups, 73.3% of the participants were of normal weight, 12.5% were pre-obese, 11.7% were underweight, and 2.5% were obese. The mean age of the participants was 22.01, with ages ranging from 18.00 to 32.00. While the average height was 168.59 cm (min: 154.00 cm; max: 188.00 cm), the average weight was 62.61 kg (min: 43.00 kg; max: 98.00 kg) (Table 1).

**Table 1:** Demographic variables.

Variable		Groups		f		%	
**Gender**		Woman		63		52,5	
		Man		57		47,5	
**Grade**		1st		9		7,5	
		2nd		19		15,8	
		3rd		77		64,2	
		4th		15		12,5	
**Daily meal amount**		1		3		2,5	
		2		49		40,8	
		3		56		46,7	
		4 and more		12		10,0	
**BMI Groups**		Underweight		14		11,7	
		Normal		88		73,3	
		Owerweight		15		12,5	
		Obese		3		2,5	
**Min.**		**Max.**		**Mean**		**SD.**	
**Age**	18,00		25,00		22,01		1,775
**Height (cm)**	154,00		188,00		168,59		7,995
**Weight (kg)**	43,00		98,00		62,61		10,935

Descriptive statistics for the Bigorexia scale and other measurements presented in Table 2 reveal the general structure of the data. The mean scores for the Bigorexia scale and its subscales were 36.0250 (sd = 9.17749) for Bigorexia, 10.9000 (sd = 3.06622) for Functional Impairment, 13.3417 (sd = 3.53731) for Volume Work, and 11.7833 (sd = 3.22590) for Appearance Intolerance, respectively. The participants’ mean BDNF, Asprosin, and GLP1 levels were measured as 10.3422, 10.4803, and 120.0548, respectively. The internal consistency coefficients of the scales are 0.854 for the Bigorexia scale, 0.765 for Functional impairment, 0.787 for volume work, and 0.834 for Appearance intolerance (Table 2). According to the Independent Samples T-Test results, men had significantly higher mean scores on bigorexia and its subscales (functional impairment, working for volume, and appearance intolerance) than women (*p* < 0.05). However, no significant differences were found in BDNF, Asprosin, and GLP1 levels by gender (Table 3), (*p* > 0.05). No statistically significant differences were found between the BMI groups in terms of Bigorexia, Functional Impairment, Volumetrics, BDNF, and GLP1 scale and measurement scores. However, significant differences were found in Appearance Intolerance scores (*p* = 0.048) and Asprosin levels (*p* = 0.008). Appearance Intolerance scores were significantly higher in lean participants than in obese participants, and Asprosin levels were significantly higher in the obese group than in the healthy group (Table 4).

**Table 2:** Descriptive statistics of the bigorexia scale and other measurements.

Scale	Min.	Max.	$\:\stackrel{-}{\varvec{X}}$	s	Skewness	Kurtosis	Cronbach Alfa
Bigorexia	17,00	63,00	36,0250	9,17749	-,017	-,319	0,854
Functional disorder	4,00	20,00	10,9000	3,06622	-,061	-,266	0,765
Working for volume	6,00	24,00	13,3417	3,53731	,077	-,123	0,787
Appearance intolerance	5,00	19,00	11,7833	3,22590	-,050	-,757	0,834
BDNF	6,24	14,32	10,3422	2,14406	,233	-,814	-
Asprosin	6,75	18,62	10,4803	2,51948	,634	,937	-
GLP1	90,76	138,22	120,0548	14,38301	-,743	-,834	-

**Table 3:** Comparison of bigorexia scale and other measurement scores by gender (Independent samples T-Test).

Parameters	Gender	*N*	$\:\stackrel{-}{\varvec{X}}$	s	t	sd	*p*
**Bigorexia**	Woman	63	31,7460	7,43568	−6,143	118	,000
	Man	57	40,7544	8,62571			
**Functional disorder**	Woman	63	9,5079	2,55818	−5,932	118	,000
	Man	57	12,4386	2,85368			
**Working for volume**	Woman	63	11,8254	2,92122	−5,513	118	,000
	Man	57	15,0175	3,41996			
**Appearance intolerance**	Woman	63	10,4127	2,72169	−5,452	118	,000
	Man	57	13,2982	3,07620			
**BDNF**	Woman	63	10,3438	2,28722	,009	118	,993
	Man	57	10,3404	1,99417			
**Asprosin**	Woman	63	10,3073	2,51170	-,790	118	,431
	Man	57	10,6716	2,53644			
**GLP1**	Woman	63	120,0363	14,94758	-,015	118	,988
	Man	57	120,0751	13,86475			

**Table 4:** Comparison of bigorexia scale and other measurement scores with BMI groups (ANOVA).

Parameters	BKİ	*N*	$\:\stackrel{-}{\varvec{X}}$	s	F	*p*	Post Hoc (Tukey)
**Bigorexia**	Underweight	14	38,7143	9,46596	2,588	,079	-
	Normal	88	36,4432	8,96206			
	Obese	18	31,8889	9,22203			
**Functional disorder**	Underweight	14	11,7857	2,86030	2,294	,105	-
	Normal	88	11,0227	3,05496			
	Obese	18	9,6111	3,05130			
**Working for volume**	Underweight	14	13,9286	3,91180	1,619	,105	-
	Normal	88	13,5227	3,53952			
	Obese	18	12,0000	3,06786			
**Appearance intolerance**	Underweight	14	13,0000	3,35123	3,119	,048	a > c
	Normal	88	11,8977	3,06647			
	Obese	18	10,2778	3,52813			
**BDNF**	Underweight	14	10,5943	1,92560	,159	,853	-
	Normal	88	10,2785	2,28117			
	Obese	18	10,4572	1,62026			
**Asprosin**	Underweight	14	10,1779	2,33946	5,032	,008	c > b
	Normal	88	10,1847	2,19822			
	Obese	18	12,1611	3,46420			
**GLP1**	Underweight	14	118,4057	14,09489	,166	,847	-
	Normal	88	120,4990	14,55754			
	Obese	18	119,1656	14,40614			

According to the results of the Independent Samples T-Test, a significant difference was found in BDNF levels based on the number of daily meals. BDNF levels were higher in those who consumed 1–2 meals per day than in those who consumed 3 or more meals (*p* = 0.035). No significant differences were found in the Bigorexia scale and its subscales, as well as in Asprosin and GLP1 levels (Table 5), (*p* > 0.05).

**Table 5:** Comparison of bigorexia scale and other measurement scores with the number of daily meals (Independent samples T-Test).

Parameters	Meal per day	*N*	$\:\stackrel{-}{\varvec{X}}$	s	t	sd	*p*	
**Bigorexia**	1–2	52	35,5577	8,55756	-,486	118	,628	
	3+	68	36,3824	9,67229				
**Functional disorder**	1–2	52	10,7308	3,07498	-,527	118	,118	
	3+	68	11,0294	3,07601				
**Working for volume**	1–2	52	13,0385	3,33115	-,820	118	,414	
	3+	68	13,5735	3,69476				
**Appearance intolerance**	1–2	52	11,7885	2,91942	,015	118	,988	
	3+	68	11,7794	3,46344				
**BDNF**		1–2	52	10,8125	2,18874	2,132	118	,035
		3+	68	9,9825	2,05311			
**Asprosin**		1–2	52	10,8637	2,68061	1,464	118	,146
		3+	68	10,1872	2,36716			
**GLP1**		1–2	52	121,1848	13,43737	,751	118	,454
		3+	68	119,1906	15,10745			

Correlation analysis examining the relationships between participants’ continuous demographic variables, the Bigorexia Scale, and BDNF, Asprosin, and GLP1 levels revealed a significant positive correlation between height and weight (*r* = 0.501; *p* < 0.01). There was also a very strong positive correlation between weight and BMI (*r* = 0.847; *p* < 0.01). Significant positive correlations were found between height and the Bigorexia Scale and all of its subscales (Functional Impairment, Working for Volume, and Appearance Intolerance). However, significant negative correlations were found between BMI and the Bigorexia Scale and its subscales (*p* < 0.01). All of the Bigorexia Scale and its subscales were found to be positively and highly correlated with each other (*p* < 0.01). When biomarkers were examined, significant positive correlations were found between weight and BMI and Asprosin (*r* = 0.265 and *r* = 0.290, respectively; *p* < 0.01). There was also a strong positive correlation between asprosin and GLP1 levels (*r* = 0.585; *p* < 0.01). No significant correlation was found between age and BDNF variables in any measurement (Table 6), (*p* > 0.05).

**Table 6:** Relationships between continuous demographic variables of the participants, bigorexia scale, and BDNF, Asprosin, and GLPI levels (Correlation Analysis).

		1	2	3	4	5	6	7	8	9	10	11
**1.Age**	r	1										
**2.Height (cm)**	r	-,119	1									
**3.Weight (kg)**	r	-,038	,501^**^	1								
**4.BMI**	r	,027	-,028	,847^**^	1							
**5.Bigorexia**	r	-,011	,366^**^	-,085	-,320^**^	1						
**6. Functional disorder**	r	,036	,322^**^	-,095	-,310^**^	,949^**^	1					
**7. Working for volume**	r	-,108	,390^**^	-,047	-,286^**^	,943^**^	,871^**^	1				
**8.Appearance intolerance**	r	,053	,308^**^	-,099	-,303^**^	,909^**^	,794^**^	,758^**^	1			
**9.BDNF**	r	-,036	,016	-,040	-,067	,048	,039	,091	-,002	1		
**10.Asprosin**	r	,027	,039	,265^**^	,290^**^	-,012	-,005	-,034	,008	,071	1	
**11.GLP1**	r	-,021	,045	,116	,104	,081	,116	,042	,075	,132	,585^**^	1

The majority of the participants (64.2%) were third-year students, and 52.5% were female. The mean age was 22.01 years, height was 168.59 cm, and weight was 62.61 kg. The mean bigorexia scale score was determined as 36.0250 (sd = 9.17749). According to the results of the Independent Samples T-Test, men had statistically significantly higher bigorexia scale and subscale scores than women (*p* < 0.05). Analysis of variance between BMI groups revealed significant differences in Appearance Intolerance (*p* = 0.048) and Asprosin levels (*p* = 0.008). Chi-Square analysis showed a significant relationship between bigorexia status and consumption of meat and meat products (*p* = 0.000) and fat consumption (*p* = 0.025). No significant differences or relationships were found for other variables and scales (*p* > 0.05), (Table 7).

**Table 7:** Relationships between participants’ bigorexia status and their product consumption in the last month (Chi-Square Analysis).

		Bigorexia status					
Food Frequency		Low	High	Total	X^2^	sd	*p*
**Milk-Yogurt**	Always	2	3	5	3,449	4	,486
	5–6 times a week	12	9	21			
	3–4 times a week	26	17	43			
	1–2 times a week	22	18	40			
	Never	9	2	11			
**Meat-Meat product**	Always	0	5	5	48,483	4	,000
	5–6 times a week	10	27	37			
	3–4 times a week	21	16	37			
	1–2 times a week	31	0	31			
	Never	9	1	10			
**Egg**	Always	5	7	12	6,978	4	,137
	5–6 times a week	15	7	22			
	3–4 times a week	20	22	42			
	1–2 times a week	20	9	29			
	Never	11	4	15			
**Bread and Cereals**	Always	23	22	45	5,761	4	,218
	5–6 times a week	9	7	16			
	3–4 times a week	18	13	31			
	1–2 times a week	16	7	23			
	Never	5	0	5			
**Vegetables**	Always	8	1	9	6,607	4	,158
	5–6 times a week	11	8	19			
	3–4 times a week	23	17	40			
	1–2 times a week	21	21	42			
	Never	8	2	10			
**Fruits**	Always	12	7	19	1,158	4	,885
	5–6 times a week	17	13	30			
	3–4 times a week	23	19	42			
	1–2 times a week	14	8	22			
	Never	5	2	7			
**Fats-Oils**	Always	6	6	12	11,1333	4	,025
	5–6 times a week	8	1	9			
	3–4 times a week	14	16	30			
	1–2 times a week	27	23	50			
	Never	16	3	19			
**Sugar and Candy**	Always	13	11	24	2,830	4	,587
	5–6 times a week	11	3	14			
	3–4 times a week	15	13	28			
	1–2 times a week	24	17	41			
	Never	8	5	13			

A one-way analysis of variance (ANOVA) was conducted to determine the differences between participants’ Body Mass Index (BMI) groups and Asprosin, GLP-1, and BDNF levels. Asprosin levels were found to differ statistically significantly among BMI groups (Normal, Overweight/Pre-obese, Underweight, Obese), F(3, 107) = 4.295, *p* = 0.007. The strength of this effect was found to indicate a moderate effect size. Post-hoc Bonferroni tests showed that mean Asprosin levels were significantly higher in the Obese group than in the Healthy (Normal Weight) group (*p* = 0.008). GLP-1 levels were not found to differ statistically significantly between BMI groups, F(3, 107) = 1.107, *p* = 0.348. The effect size for this relationship was small/negligible, with ηp2 = 0.030. A significant difference was found in the comparison between the groups with the number of daily meals (1–2 meals, 3 meals, ≥ 4 meals) and BDNF levels, F(2, 107) = 3.511, *p* = 0.033. This effect had a moderate effect size of ηp2 = 0.081. Post-hoc analyses revealed that participants who consumed 1–2 meals per day had higher mean BDNF levels than the other groups.

Research findings showed that BGN scale scores significantly influenced participants’ performance on the 6-minute walk test (6MWT) (*p* = 0.004 < 0.01); specifically, individuals with higher BGN levels (mean 748.45 m) walked a greater distance in 6 min than individuals with lower BGN levels (mean 733.19 m). However, BGN scale scores did not significantly influence expected 6MWT (*p* = 0.453), walking speed (*p* = 0.459), or metabolic equivalent (MET) (*p* = 0.510); that is, there were no significant differences between the low and high BGN groups (*p* > 0.05) (Table 8).

**Table 8:** Participants’ 6-meter walking test, speed, and metabolic equivalent data according to their bigorexia status.

Parameters	Bigorexia Level	*N*	Mean	Std. Deviation	t	df	*p*
**Expected 6MWT**	Low BGN	71	740,4970	46,56600	,754	118	,453
	High BGN	49	734,2829	41,01911			
**6MWT realized**	Low BGN	71	733,1887	27,28143	−2,959	118	0,004**
	High BGN	49	748,4510	28,47853			
**Speed**	Low BGN	71	123,4006	7,75732	,742	118	,459
	High BGN	49	122,3806	6,83670			
**MET**	Low BGN	71	4,4996	,22,647	,661	118	,510
	High BGN	49	4,4729	,20,373			

**p < 0.01,* *p* < 0.05; Low BGN (Low Bigorexia Nervosa Low): Those with a Bigorexia Nervosa Scale Mean Score of < 39, High BGN (High Bigorexia Nervosa High): Those with a Bigorexia Nervosa Scale Mean Score of ≥ 39.

## Discussion

BGN is a body dysmorphic disorder characterized by a preoccupation with insufficient muscle mass. Groups such as bodybuilders, weightlifters, adolescent and young adult college students, men in their twenties, coaches, and models are particularly at risk due to heightened body image sensitivity and social comparisons^29,30^.Understanding the sociodemographic characteristics of BGN is critical for identifying risk groups, especially young male athletes and university students, to prevent it and develop effective treatment strategies^30–32,33^.Bigorexic individuals consider excessive exercise and intense, high-protein, low-fat diets aimed at muscle building as normal behavior^1^.This study shows that men have higher BGN levels than women (*p* < 0.05) and that BGN is associated with eating habits. It was also found that BGN is positively correlated with height and negatively correlated with Body Mass Index (BMI) (*p* < 0.01); these results are consistent with the literatüre^1–3,29,32^. Our results highlight that men are at higher risk for BGN than women, and this is associated with factors such as eating habits, height, and body mass index (BMI).

In this study, the mean score of students on the Bigorexia Inventory was found to be 36.02 ± 9.17, and 40.8% of students were found to have a tendency towards bigorexia. Mean Bigorexia Inventory scores vary across studies. White et al.^34^ reported a mean of 29.13 ± 8.83, while Bégin et al.^35^ found a lower mean of 19.48 ± 8.41. The prevalence of bigorexia tendency also varies across studies; Compte et al.^36^ found this tendency in 6.99% of male university students, while Longobardi et al.^37^ found this rate to be 25%. This difference between studies is thought to be due to the fact that the studies were conducted with different populations.

Although BGN is considered a psychological disorder, hormones are reported to be related to the development or symptoms of bigorexia^2^. Asprosin is effective in adipose tissue regulation with metabolic response^38,39^. GLP-1, an incretin hormone secreted from the intestines, is effective in appetite control, satiety and mood regulation^40^ and BDNF is effective in brain neuronal plasticity and mood regulation^14,41^. Furthermore, CREB levels, a central factor in the transcriptional activation of BDNF, are closely associated with neuronal plasticity and associated mood regulation^42^.According to the study results, no direct significant relationship was found between bigorexia and asprosin levels (*p* > 0.05). Significant differences were found between asprosin levels and BMI groups (*p* = 0.008). Specifically, asprosin levels were significantly higher in the obese group compared to the normal-weight group (*p* < 0.05). Furthermore, positive correlations were observed between weight and BMI and asprosin (*r* = 0.265 and *r* = 0.290, respectively; *p* < 0.01). Given the unhealthy eating habits and changes in body composition in individuals with body dysmorphic disorders such as bigorexia, examining metabolic markers such as asprosin is important. Although no direct relationship was found between bigorexia and asprosin in this study, its significant relationship with obesity and BMI indicates that asprosin maintains its importance as an indicator of metabolic health. Our current results are consistent with the results reporting a significant relationship between asprosin hormone and obesity and BMI^43–46^.

Despite the known effects of GLP-1 on general appetite control, satiety, and mood by reducing glucagon levels and increasing insulin levels, no significant relationship was found between BGN levels and GLP-1 or asprosin levels (*p* > 0.05). The fact that GLP-1 levels were not affected by factors such as gender (*p* = 0.988), number of meals per day (*p* = 0.454), and BMI (*p* = 0.847) suggests that the GLP-1 data in this study were relatively homogeneous across the population and that these demographic or physiological variables did not have a significant effect on GLP-1 levels. This finding supports the need to examine the relationship between GLP-1 and bigorexia through other factors. The strong positive correlation between asprosin and GLP-1 (*r* = 0.585; *p* < 0.01) suggests that these two hormones may share a common regulatory pathway or interact with each other in energy metabolism, appetite, and body composition. Although our results show that GLP-1 has no direct relationship with BGN, its strong interaction with asprosin is consistent with research results reporting its role in appetite regulation^47–49^.

The study examined BDNF levels, which play an important role in stress-related psychological discomfort and disorders, and revealed no significant gender-related differences in mean salivary BDNF, asprosin, and GLP-1 levels (*p* > 0.05). Participants who ate 1–2 meals per day had significantly higher salivary BDNF levels than those who ate 3 or more meals per day (*p* = 0.035), suggesting that meal frequency may affect BDNF levels. While no correlation was found between age and bigorexia and BDNF levels (*p*=−0.036 and *p* = 0.048, respectively), meal frequency affected BDNF (*p* = 0.035), but no significant correlation was found between BDNF and BGN levels (*p* = 0.048). While this finding contradicts research findings reporting an increase in psychological distress reported in the literature^50,51^, it is consistent with similar research findings^52,53^.This finding is thought to stem from methodological differences such as sample size and factors related to physiological processes such as the severity and duration of BGN. Because psychological distress such as anxiety and depression negatively impact individuals’ physical activity levels, the Six-Minute Walk Test (6MWT) and MET can be used as indicators of decreased functional capacity or exercise intolerance^54,55^. In our current study, it was found that BGN levels significantly affected participants’ 6MWT performance (*p* = 0.004 < 0.01), with individuals with higher BGN levels (mean 748.45 m) walking further than those with lower BGN levels (mean 733.19 m). However, BGN scores did not significantly affect expected 6MWT, walking speed, or metabolic equivalent (MET) values, which were found to be 4.53 on average (*p* > 0.05). Our current results are similar to the results of similar studies reported in the literature that although the 6-Minute Walk Test (6MWT) and Metabolic Equivalent (MET) values are not direct diagnostic tools for bigorexia, they can be useful in evaluating the exercise capacity of individuals with psychological disorders and monitoring treatment responses^22,56–58^.

There are several important limitations that should be considered when interpreting the results of this study. First, the cross-sectional design of our study precludes establishing a cause-and-effect relationship between BGN levels and hormone levels (asprosin, GLP-1, and BDNF). Future longitudinal studies are needed to determine the direction of causality and time course of the observed relationships. Second, the use of self-report questionnaires to assess BGN and dietary habits may introduce social desirability bias in the results and may weaken the strength of associations with physiological markers by influencing the true level of BGN symptoms or dietary patterns. Another limitation is that the sample was limited to students in the Faculty of Sports Sciences, limiting the generalizability of the results to the general athlete population or higher-risk groups such as professional bodybuilders. Finally, biomarkers such as asprosin, GLP-1, and BDNF are influenced by numerous confounding variables, such as circadian rhythms, stress levels, and the timing of physical activity. A single, point-in-time measurement may not fully reflect daily fluctuations in these hormones. Therefore, the use of repeated biological measurements in the future is recommended to increase the reliability of our findings.

## Conclusion

The data obtained in our current study determined that men have higher levels of Bigorexia Nervosa (BGN) than women. Furthermore, BGN is associated with eating habits such as meat, meat products, and fat consumption. Based on these results, it is recommended that male athletes and bodybuilders, in particular, be considered at-risk groups for obsession with muscle mass and unhealthy eating behaviors, and that counseling and education programs be developed accordingly. Furthermore, examining endocrine (asprosin, GLP-1) and cerebral (BDNF) hormone levels in individuals with BGN will contribute to the practices, programs, and strategies of dietitians, sports psychologists, and sports physicians to provide effective multidisciplinary approaches and support individuals’ psychological and physiological well-being. However, as this study is one of the first to examine the relationship between BGN and hormonal levels, further research is needed to fully elucidate the mechanism of this relationship. We also anticipate that levels of hormones such as asprosin and BDNF may serve as early indicators and biomarkers for addressing risk factors associated with clinical conditions closely associated with BGN, such as eating disorders, anxiety disorders, and body dysmorphic disorders. Consequently, given the health consequences of body image issues and unhealthy eating habits, this study is expected to be particularly instructive for the fields of sports dietetics, sports psychology, and public health.

## Data Availability

The corresponding author upon reasonable request will provide data supporting the findings of this study.
